# Targeting the UPS as therapy in multiple myeloma

**DOI:** 10.1186/1471-2091-9-S1-S1

**Published:** 2008-10-21

**Authors:** Dharminder Chauhan, Giada Bianchi, Kenneth C Anderson

**Affiliations:** 1 The Jerome Lipper Multiple Myeloma Center, Department of Medical Oncology, Dana Farber Cancer Institute, Harvard Medical School, Boston, MA 02115, USA

## Abstract

The coordinated regulation of cellular protein synthesis and degradation is essential for normal cellular functioning. The ubiquitin proteasome system mediates the intracellular protein degradation that is required for normal cellular homeostasis. The 26S proteasome is a multi-enzyme protease that degrades redundant proteins; conversely, inhibition of proteasomal degradation results in intracellular aggregation of unwanted proteins and cell death. This observation led to the development of proteasome inhibitors as therapeutics for use in cancer. The clinical applicability of targeting proteasomes is exemplified by the recent FDA approval of the first proteasome inhibitor, bortezomib, for the treatment of relapsed/refractory multiple myeloma. Although bortezomib represents a major advance in the treatment of this disease, it can be associated with toxicity and the development of drug resistance. Importantly, extensive preclinical studies suggest that combination therapies can both circumvent drug resistance and reduce toxicity. In addition, promising novel proteasome inhibitors, which are distinct from bortezomib, and exhibit equipotent anti-multiple myeloma activities, are undergoing clinical evaluation in order to improve patient outcome in multiple myeloma.

Republished from Current BioData's Targeted Proteins database (TPdb; ).

## Protein pathway involvement in disease

### The ubiquitin proteasome system (UPS)

Intracellular protein degradation occurs primarily through a multisubunit complex called the proteasome [1-3]. Pioneering studies by Ciechanover *et al. *showed that ATP-dependent conjugation of proteins with a polypeptide, ubiquitin (UBIQ), is required for protein degradation [4-7]. Subsequent reports demonstrated the role of UBIQ in cellular protein turnover [8,9], and it is now well established that ubiquitylation of proteins accounts for their stability, functionality, localization and interactive capabilities [10].

UBIQ-mediated proteolysis occurs via the 26S proteasome complex [11-15], which contains 19S units flanking a barrel-shaped 20S proteasome core [16,17]; the 19S units regulate entry of ubiquitylated proteins into the 20S core chamber [2,18,19]. Protein breakdown involves sequential enzymatic reactions that culminate in target proteins becoming linked to a chain of UBIQ molecules. In the first reaction, the E1 ubiquitin enzyme activates UBIQ and attaches it to the ubiquitin-conjugating enzyme E2 in an ATP-dependent manner. The E3 ubiquitin ligase then links the UBIQ molecule to the target protein or to a previously attached UBIQ moiety [20]. Sequential cycles of this process lead to the formation of polyubiquitylated proteins that are eventually degraded by the proteasomes into small peptides, with re-cycling of free UBIQ [21-24]. Importantly, E3 ubiquitin ligases confer specificity in the UBIQ signaling pathway by selectively targeting potential protein substrates for ubiquitylation and subsequent proteasomal degradation [25]. Three proteasomal activities that regulate proteolysis are chymotrypsin-like (CT-L), trypsin-like (T-L) and caspase-like (C-L), also known as β5, β2 and β1, respectively; all of these reside within the 20S proteasome core [26-28].

Proteolysis is a normal cellular process and thus substrates for proteasomes include many cellular proteins that maintain normal cell cycle progression, growth and survival [1,2,8,29-31]. Conversely, pharmacological inhibition of proteasome function hampers the normal elimination of misfolded proteins, thereby causing a build-up of unwanted proteins and eventual cell death [32-34]. These laboratory observations have translated into the clinical application of proteasome inhibitors as anticancer therapies. However, since the proteasome regulates normal cellular functions, its inhibition could also trigger toxicity against normal cells [33,35,36]. Importantly, recent studies have shown that proteasome inhibitors are more cytotoxic to proliferating malignant cells than quiescent normal cells, suggesting a favorable therapeutic index [35-41].

### Targeting the UPS in multiple myeloma

As stated in the section *The ubiquitin proteasome system (UPS)*, a broad spectrum of intracellular proteins are substrates for proteasome-mediated degradation. This process involves NFκB, a transcription factor that plays a pivotal role in the inflammatory response and carcinogenesis by controlling genes involved in growth, survival, cell cycle progression, angiogenesis and invasion [42-44]. Palombella *et al. *showed that the UPS is required for processing the NFKB1 (NFκB1) precursor protein and for activation of NFκB [45]. Conversely, inhibition of proteasomes by proteasome inhibitor MG132 blocked NFκB activity [46]. Importantly, our studies showed that adhesion of MM cells to bone marrow stromal cells (BMSCs) triggered transcription and secretion of various cytokines that confer growth, survival and drug resistance in MM cells [47,48]. Other studies further confirmed the role of NFκB as a major growth and survival signaling pathway in MM [49-53]. The survival- and growth-promoting role of NFκB in MM, together with the ability of proteasome inhibitors to block NFκB, provided the initial rationale for proteasome inhibitor therapy in MM. Studies by Palombella *et al. *focused on the development of a series of peptide boronic acid inhibitors of the proteasome, in particular bortezomib, and demonstrated their inhibitory activity on NFκB [46]. The dipeptidyl boronic acid bortezomib is a potent and reversible inhibitor of the CT-L activity of the proteasome [33,41]. Initial NCI screening showed the remarkable antitumor activity of bortezomib against a panel of 60 tumor cell lines [41,54]. Our *in vitro *studies showed that bortezomib downregulates NFκB, blocks constitutive and MM cell adhesion-induced cytokine secretion in BMSCs and induces apoptosis in both MM cell lines and freshly isolated MM cells from patients [35,51]. Moreover, *in vivo *studies using animal models (see *Disease models*, *knockouts*, *assays*) demonstrated a potent anti-MM activity of bortezomib [36,55].

## Disease models, knockouts, assays

Our *in vitro *findings have also been validated *in vivo *using animal models. These models represent an indispensable bridge between basic research and clinical drug development and have been extensively used in the validation of bortezomib and other novel compounds against MM [55-57]. One of the first models to be used was the human plasmacytoma xenograft model, in which triple immunodeficient mice (beige-nude-xid) were injected subcutaneously with MM cells [55]. This model allows us to simultaneously measure the effect of *in vivo *drug treatment on the growth of human MM cells (assessed by tumor cell size, human idiotypic protein in mouse serum and host survival), as well as the neighboring blood vessels (assessed by microvessel density). Our studies using this model showed that bortezomib inhibits both human MM cell growth and associated angiogenesis *in vivo *[55].

Although the human plasmacytoma xenograft model allows for direct assessment of therapy of tumor growth and associated angiogenesis, it does not reflect the bone marrow milieu. For these studies, we have utilized the SCID-hu mouse model developed in our laboratory [58]. This model examines MM cell growth in the human bone marrow milieu, which includes human BMSCs, extracellular matrix proteins and cytokines, and best reflects the human disease [58-60]. In this model, MM cells are injected directly into human fetal bone chips implanted subcutaneously in SCID mice. Tumor growth is assessed by evaluating circulating levels of soluble human IL6R and human monoclonal antibody levels using ELISA [57]. This model allows us to perform immunohistochemical analysis of excised human bone chips in order to determine MM cell growth *in vivo *using staining with antibodies specific for tumor cells (SYND1 [CD138]). Immunohistochemical analysis of human bone sections from mice treated with various drugs, such as bortezomib, lenalidomide or NPI-0052, can be performed to detect apoptosis (CASP3 [caspase-3] activity, TUNEL staining), growth inhibition (Ki67) and angiogenesis (PECA1 [CD31] and FA8 [Factor VIII staining]). ELISA and human cytokine bead arrays are utilized for analyzing the effect of drugs on human cytokine secretion in mouse serum triggered by human MM-BMSC interactions. This model therefore permits the evaluation of the molecular and cellular changes induced by human MM-human stroma interactions *in vivo*, before and after therapy. We have used this model to validate novel targeted therapies such as CD-138-DM1 antibody [57] and BAFF inhibitor [61]. Our ongoing studies are examining the effects of various proteasome inhibitors in this model.

Validation of the proteasome as a target for antineoplastic therapy has led to the development of assays that not only quantify the bulk of the proteasomes in cells, but also qualitatively assess their function [3,28]. Given the differential proteolytic activities of the proteasome and the growing number of available inhibitors, it is essential to establish a relationship between the effectiveness of the drugs and the extent and type of proteasomal blockade. Fluorogenic peptide substrates specific for each proteasomal subunit provide a rapid, sensitive and accurate tool for assessing residual proteasomal activity in crude lysates [62]. Although extremely useful, these probes require prior cell lysis, thus isolating the proteasome from its functional, regulatory environment [63]. Moreover, fluorogenic substrates do not differentiate between constitutive and interferon-inducible (immunoproteasome) proteasomal activities [63]. These limitations have aroused interest in designing cell permeable, specific proteasome probes, such as DansylAhx_3_L_3_VS, capable of monitoring proteasome activity in living cells [64]. DansylAhx_3_L_3_VS has been proven to be useful for defining the differential activity profiles of the two proteasome inhibitors bortezomib and NPI0052 in MM cell lines [36]. It was also effective in differentiating between the inhibition of constitutive and inducible proteasomal activity in peripheral blood mononuclear cells from patients undergoing treatment with bortezomib [65]. These experiments confirm the use of DansylAhx_3_L_3_VS for quantitatively and qualitatively monitoring proteasomal blockade in patients receiving proteasome inhibitor therapy [65].

## Disease targets and ligands

Preclinical studies provided the basis for the evaluation of bortezomib as a therapy in MM. A Phase I clinical trial on 27 patients with refractory hematologic malignancies showed anti-MM activity and acceptable toxicity, and in a Phase II trial enrolling 202 relapsed, refractory MM patients, one third of the patients achieved durable responses (with 4% complete responses) and associated clinical benefit. This provided the basis for accelerated FDA approval of bortezomib treatment for relapsed refractory MM [66,67]. Thereafter, a randomized Phase III trial on 669 MM patients who had relapsed after at least one prior treatment showed higher responses, as well as prolonged time to progression and survival, after treatment with bortezomib when compared with dexamethasone [68]. This provided the basis for the extension of FDA approval of bortezomib to include treatment of relapsed MM. Despite the success of bortezomib therapy (43% objective response rates), it has been associated with possible off-target toxicities and the development of drug resistance [67,69-72]. Therefore, extensive laboratory efforts are now focused on delineating the molecular mechanisms mediating bortezomib cytotoxicity and drug resistance, in order to both enhance sensitivity and overcome clinical resistance to bortezomib.

### Mechanistic insights into bortezomib-induced cell death

Bortezomib-triggered apoptosis in MM cells is associated with inhibition of NFκB; however, whether NFκB blockade is an obligatory event remains unclear. To address this issue, we compared the effects of PS1145, a specific inhibitor of IKKB (IKK-B), with the effects of bortezomib on NFκB and consequent biological response (cell death) in MM cells. Both PS1145 and bortezomib block TNFA (TNF-α)-induced NFκB activation by inhibiting phosphorylation and degradation of IKKB. However, in contrast to bortezomib, PS1145 only partially inhibits MM cell growth [50]. These findings showed that NFκB inhibition alone is unlikely to account for the overall anti-MM activity of bortezomib. Supportive of these findings, studies to date suggest that bortezomib affects both growth and apoptotic signaling pathways. For example, bortezomib-triggered apoptosis is associated with: activation of heat shock proteins (HSPB1 [Hsp27], Hsp70 and Hsp90) [73,74]; upregulation of JNK/SAPK [73,75,76]; generation of reactive oxygen species [77]; release of CYC (cytochrome-c)/DBLOH (Smac) from mitochondria into the cytosol and activation of CASP9 (caspase-9) and CASP3 (caspase-3) [75,76]; activation of BIM (Bim) and CASP8 (caspase-8) [73,76]; blockade of DNA repair [78]; inhibition of MM-BMSC interactions and sequelae including activation of MAPK and PI3K signaling pathways [35,79], and induction of ER stress and an unfolded protein response [80,81]. Together, these *in vitro *studies suggest that proteasome inhibition by bortezomib triggers both various apoptotic signaling cascades and blocks growth/survival mechanisms in MM cells [82].

## New frontiers in drug discovery

Oncogenomics and proteomic studies in MM cells are identifying and validating key molecules, the pharmacological inhibition of which enhances the antitumor activity of bortezomib and could abrogate drug resistance [83]. Firstly, bortezomib induces Hsp90 in MM cells, whereas blockade of Hsp90 with 17AAG (Kosan Biosciences, USA) enhances sensitivity and even overcomes resistance to bortezomib [84]. Ongoing clinical trials show that combination therapy can achieve responses in refractory MM [85]. Secondly, laboratory studies show that bortezomib induces HSPB1 (Hsp27), and MM cells from patients resistant to bortezomib overexpress HSPB1 [74,86]. These findings have already been tested in a clinical trial by combining bortezomib with a p38 MAPK inhibitor, SCIOS469 (Scios USA), which downregulates HSPB1 and overcomes bortezomib resistance [87,88]. Thirdly, treatment of MM cell lines and SYND1 (CD138)^+ ^MM cells with bortezomib and the novel agents lenalidomide (immunomodulatory agent from Celgene, USA) or CDDO-Imidazolide (a triterpenoid initially synthesized by Honda *et al., *Dartmouth College, USA) induces synergistic anti-MM activity *in vitro *and overcomes resistance to bortezomib by targeting both intrinsic and extrinsic apoptotic signaling. This was evidenced by the disruption of mitochondrial potential and cleavage of CASP9 (caspase-9) and CASP8 (caspase-8) [89,90]. These *in vitro *studies provide the basis for clinical protocols combining these agents [91]. Fourth, bortezomib combined with conventional anti-MM agents such as dexamethasone (Merck USA), doxorubicin (Pharmacia S.p.A, Italy), melphalan (GlaxoSmithKline, UK) or mitoxantrone (Lederle Parenterals, Puerto Rico) induces additive or synergistic antitumor activity in MM cell lines and freshly isolated SYND1^+ ^MM cells, as measured by decreased cell viability in colorimetric assays (MTT assay) [78].

A recent randomized Phase III trial in 646 patients showed that treatment with pegylated doxorubicin and bortezomib achieved increased overall survival and extent of responses, as well as prolonged time to progression, compared with bortezomib alone. This provided the rationale for FDA approval of this combination for the treatment of relapsed MM [92,93]. Finally, our preclinical studies showed that the simultaneous targeting of lysosomal and proteasomal (non-lysosomal mechanism) protein degradation triggers synergistic anti-MM activity [94,95]. For example, HDAC6 (HDAC-6) is required for the chaperoning of ubiquitylated proteins for aggresomal degradation. Simultaneous inhibition of proteasomes and aggresomes with bortezomib and tubacin (HDAC6 inhibitor) induces synergistic cytotoxicity in MM cell lines and primary cells from MM patients [95]. A recent study in pancreatic cancer cells showed that bortezomib triggered the formation of aggresomes (aggregates of ubiquitin-conjugated proteins), which are cytoprotective, whereas their disruption by HDAC6 siRNA induced synergistic apoptosis [96]. These findings set the stage for the evaluation of combined treatment protocols of bortezomib with HDAC inhibitors in both hematologic malignancies and solid tumors. Our study has already validated the anti-MM activity of HDAC inhibitor LBH589 (Novartis Pharmaceuticals, USA) [97]; and a Phase I safety study of LBH589, given in combination with bortezomib, is ongoing in MM patients [98].

### Discovery and validation of novel proteasome inhibitors NPI0052 and PR171 as therapy

A recent study showed that a novel proteasome inhibitor, NPI0052 (salinosporamide A), is able to overcome bortezomib resistance in MM cells. NPI0052 is a small molecule derived from the fermentation of *Salinospora*, a marine gram-positive actinomycete [36,99,100]. NPI0052 is a nonpeptide proteasome inhibitor with structural similarity to omuralide. Omuralide is clasto-lactacystin beta-lactone and is the active form of a proteasome inhibitor, lactacystin [82]. Despite the structural similarity with omuralide, NPI0052 can be distinguished by the presence of a uniquely methylated C3 RING juncture, chlorinated alkyl group at C2 and a cyclohexene ring at C5 [82,99-101]. Initial screening of NPI0052 against the NCI panel of 60 tumor cell lines showed an IC_50 _of < 10 nM in all cases. Importantly, NPI0052 similarly triggered apoptosis in purified tumor cells from several MM patients relapsing after prior therapies including bortezomib and thalidomide [36]. *In vivo *efficacy of NPI0052 was shown using a human plasmacytoma xenograft mouse model. Specifically, NPI0052 inhibited MM tumor growth and prolonged survival of these mice at concentrations that were well tolerated, without weight loss or neurological changes [36,55]. Another study showed that NPI0052 is a more effective inducer of apoptosis than bortezomib in lymphocytic leukemic cells [102].

Examination of signal transduction pathways in MM cells showed that: NPI0052 is a more potent inhibitor of NFκB and related cytokine transcription and secretion than bortezomib; NPI0052-induced MM cell death is predominantly mediated by CASP8 and bortezomib-induced apoptosis requires both CASP8 and CASP9 activation [36]. Moreover, NPI0052 and bortezomib differentially affect 20S proteasomal activities; NPI0052 inhibits all three proteasomal activities, i.e. CT-L, T-L and C-L, whereas bortezomib blocks CT-L and C-L but not T-L activities [36]. A recent study showed that simultaneous inhibition of multiple proteasome activities is a prerequisite for significant (i.e. > 50%) proteolysis [62]. Another study showed that 50% inhibition of CFTR degradation in reticulocyte extracts requires concurrent blockade of CT-L and C-L proteasome activities [103]. Whether blocking all three activities is therapeutically advantageous will be evaluated in clinical trials of NPI0052. Nonetheless, the mechanistic differences between NPI0052 and bortezomib, i.e. their differential effect on proteasome activities and their dependence on specific apoptotic signal transduction pathways, provides a rationale for combination proteasome inhibitor regimens to enhance MM cytotoxicity. Indeed, the combination of NPI0052 with bortezomib induces synergistic anti-MM activity, without significantly affecting the viability of normal lymphocytes [36]. Enhanced cytotoxicity of the combination regimen could be due to higher levels of proteasome inhibition with the two drug regimens and/or activation of differential apoptotic signaling pathways. Nevertheless, these preliminary studies set the stage for clinical trials of combined proteasome inhibitors to improve patient outcome in MM.

Finally, PR171 or carfilzomib (Proteolix, USA) is another novel proteasome inhibitor. PR171 [104-106] is an irreversible proteasome inhibitor, predominantly targeting CT-L activity and triggering potent antitumor activity [104,105]. A Phase I trial demonstrated tolerability and responses [107] and Phase II trails are ongoing in relapsed/refractory MM [108]. Laboratory studies are currently examining whether PR171 can be combined with bortezomib to induce synergistic cytotoxicity in MM cells.

## Conclusion

Delineation of the molecular and cellular mechanisms of bortezomib-induced apoptosis [79,109] has provided the rationale for combining bortezomib with conventional (dexamethasone, doxorubicin, melphalan) and novel (lenalidomide, HSP inhibitors, HDAC inhibitors) agents in order to reduce toxicity, enhance cytotoxicity and overcome drug resistance. Although bortezomib and NPI0052 belong to the same class of drugs, they are distinct in their mode of action and can be rationally combined as therapy [36,102,110]. The synergy observed in preliminary studies suggests an increased therapeutic index of combined therapy. However, more definitive evidence of enhanced efficacy of this combination regimen awaits careful clinical trials.

## List of abbreviations used

BMSC: bone marrow stromal cell; C-L: caspase-like; CT-L: chymotrypsin-like; MM: multiple myeloma; T-L: trypsin-like; UPS: ubiquitin proteasome system.

## Competing interests

The authors declare that they have no competing interests.

## Publication history

Republished from Current BioData's Targeted Proteins database (TPdb; ).
